# The Role of High-Density Lipoprotein Cholesterol in 2022

**DOI:** 10.1007/s11883-022-01012-y

**Published:** 2022-03-10

**Authors:** Cesare R. Sirtori, Alberto Corsini, Massimiliano Ruscica

**Affiliations:** Department of Pharmacological and Biomolecular Sciences, Università Degli Studi Di Milano, Milan, Italy

**Keywords:** HDL, CER-001, HDL functionality, Diabetes, Inflammation, A-I mutants, HDL therapy, Proteomics

## Abstract

**Purpose of the Review:**

High-density lipoproteins (HDL) are responsible for the transport in plasma of a large fraction of circulating lipids, in part from tissue mobilization. The evaluation of HDL-associated cholesterol (HDL-C) has provided a standard method for assessing cardiovascular (CV) risk, as supported by many contributions on the mechanism of this arterial benefit. The present review article will attempt to investigate novel findings on the role and mechanism of HDL in CV risk determination.

**Recent Findings:**

The most recent research has been aimed to the understanding of how a raised functional capacity of HDL, rather than elevated levels per se, may be responsible for the postulated CV protection*.* Markedly elevated HDL-C levels appear instead to be associated to a raised coronary risk, indicative of a U-shaped relationship.

**Summary:**

While HDL-C reduction is definitely related to a raised CV risk, HDL-C elevations may be linked to non-vascular diseases, such as age-related macular disease. The description of anti-inflammatory, anti-oxidative and anti-infectious properties has indicated potential newer areas for diagnostic and therapeutic approaches. In the last two decades inconclusive data have arisen from clinical trials attempting to increase HDL-C pharmacologically or by way of recombinant protein infusions (most frequently with the mutant A-I _Milano_); prevention of stent occlusion or heart failure treatment have shown instead significant promise. Targeted clinical studies are still ongoing.

## Introduction

High-density lipoproteins (HDL) represent approximately 25–30% of the circulating proteins responsible for carrying lipids in the circulation. HDL-cholesterol (HDL-C) is generally labeled as “good cholesterol,” although this association, evident from the earlier investigations, has not been constantly observed in recent epidemiological studies [1]. HDL has a complex structure, characterized by particles of different sizes and lipid composition, resulting in different cardiovascular protective activities. In addition to the tissue cholesterol mobilizing activity, HDL exerts a multiplicity of effects, from anti-inflammatory to anti-diabetic, anti-thrombotic, heart failure antagonism, and many others [2].

The genetic regulation of CV risk associated with HDL-C levels has been the object of intense debate. An early Mendelian randomization (MR) study [3], investigating a genetic polymorphism in the endothelial lipase (EL) gene linked to a to reduction of HDL-C, failed to detect an association with the risk of acute myocardial infarction (MI). More recently, the use of multivariate MR and MR-Egger, taking into account pleiotropy of genetic instruments (140 SNPs were considered for HDL-C), showed a modest protective effect for HDL-C on CV risk and a highly positive effect on diabetes [4]. Every 16-mg/dL increase in HDL-C led to a OR of 0.95 (95% CI, 0.85–1.06) for coronary artery disease (CAD) and of 0.83 (95% CI, 0.76–0.90) for diabetes. This finding was recently confirmed by the analysis of a large GWAS dataset from close to 300,000 individuals. Inverse variance-weighted and MR-Egger methods, evaluating pleiotropic effects of 202 SNP genetic variants for HDL-C [5••], showed an OR of 0.87 (95% CI 0.82–0.91) or of 0.88 (95%CI 0.83–0.93) for CAD, depending on the statistical test used.

While the beneficial activity of HDL-raising drugs and of infusions of HDL mimetics is still incompletely supported [6], the future of this area of research appears to be linked to potential great advances for human health. Object of this review article is a present-day critical evaluation of the huge number of clinical investigations on the protective and therapeutic roles of HDL. This will allow to elucidate potential new targets for research and therapy in the years to come.

## HDL and Prevention of Cardiovascular Risk—Development of a Concept

HDL-C remains a standard marker of CV risk. Way from the classical report by Miller and Miller [7], followed by confirmation in a Prospective Study [8], reduced HDL-C has been indicated as a major factor in raising CV risk. Many reports followed this initial observation including studies from Israel [9], the Framingham Study [10], and Sweden [11]. These initial clinical reports were rapidly followed by studies on distribution and composition of HDL subclasses, in particular evaluating conditions of extreme HDL reductions [12], as well as distribution of particles [13]. This last observation was followed by contrasting reports. While Brook et al. [9] indicated essentially identical levels of the major HDL subfractions (HDL_2_ and HDL_3_), Laakso et al. instead reported a selective reduction of HDL_2_ levels in non-insulin-dependent diabetics with coronary disease [14]. The inverse relation between HDL_2_ and coronary risk was confirmed by Robinson et al. [15], while the Framingham group indicated that the role of reduced HDL as a risk factor was particularly strong in women [16]. The inverse relation between HDL-C and coronary heart disease (CHD) was finally reported in Japanese middle-aged men [17].

While a large study in the UK did not confirm the role of low HDL-C levels as a major negative CV risk factor [18], results were criticized because of a lack of adjustment for major variables [19]. Following cross-sectional studies on HDL-C as a preventive marker of coronary risk, prospective investigations confirmed that HDL-C and the ratio of total to HDL-C provide a significant independent predictor of risk (+ 53% change in risk for a rise of one unit in this ratio) [20]. Further, the observation of an inverse relationship between HDL-C and triglycerides (TGs) prompted studies on in the catabolic pattern of apo A-I and apo A-II in patients with low HDL-C with and without hypertriglyceridemia [21], reporting that fractional catabolic rates (FCRs) of apo A-I and apo A-II have a direct correlation with plasma triglycerides (TG). In subjects with low HDL-C apo A-I is loosely bound and may be more rapidly cleared by the kidney [22].

Gender differences in HDL-C levels observed early after the beginning of epidemiological studies concluded that women have overall a 10–20% elevation of HDL-C, but this differs according to countries, the smallest differences being seen in China (2–3 mg/dL) and the largest (18–20 mg/dL) in Canada [23]. Women, in addition, are characterized by a pattern of larger HDL particle sizes, i.e., while in men, the HDL particle size is < 85 Å, the great majority of women (80%) have HDL particle sizes ⩾85 Å. HDL_2_-C levels are also 25% higher in women vs men, whereas HDL_3_-C levels are essentially identical [24]. Interestingly, however, common variants regulating cholesterol metabolism, i.e., cholesteryl ester transfer protein (CETP), hepatic lipase, lipoprotein lipase, and lecithin cholesteryl acyl transferase, appear to have a minimal impact in the variance of HDL-C and apo A-I in healthy men [25].

The outcome of coronary syndromes appears also to be influenced by HDL-C levels. In the MIRACL (Myocardial Ischaemia Reduction with Aggressive Cholesterol Lowering) trial, HDL-C levels significantly affected the short-term prognosis after acute coronary syndrome (ACS), more than LDL-C [26]. In addition, in the VA-HIT (Veterans Affairs HDL Intervention Trial) study, there was clear evidence of an association between HDL-C and events; subjects with new events had significantly lower HDL-C, apolipoprotein (apo)A-I, and large cholesterol-rich HDL particle (α-1, α-2, pre–α-1, and pre–α-2) levels, with significantly higher TG and small poorly lipidated HDL particles (pre–β-1 and α-3) vs subjects without events. The α-1 and α-2 particle levels appeared to be negative risk factors, whereas α-3 levels were a significant positive risk factor. Pre-β-1 level was a significant risk factor for new CVD events in univariate analysis vs α-1, this last apparently the best determinant of risk for recurrent events [27].

The identification of an antiatherogenic role of small dense HDL [28] prompted a series of investigations aiming to prove the link between HDL-C levels and changes in risk [29]. The start of these studies came from an investigation on nicotinic acid with the preparation of a paper by the European Consensus Panel [30], recommending that the minimum target for HDL-C should be 40 mg/dL (1.03 mmol/L) for patients with CHD or with a high risk, including patients with type 2 diabetes or the metabolic syndrome. The numerous studies on this issue provided considerable material for discussion but did not come up with significant positive contributions to therapy. In addition, a later study indicated that when apo A-I and apo B are kept constant, very high levels of HDL-C and HDL particle sizes are associated with a marked increase of CV risk [31]. This led to the early tentative conclusion of a U-shaped relationship between HDL-C and all-cause mortality [32]. This has been partly confirmed in very recent studies (see below).

The case of nicotinic acid has been also of major significance in the understanding of the still unclear correlation between HDL rises and arterial protection. Ronsay et al. [33] reported that addition of nicotinic acid to statin therapy leads to elevated levels of multiple HDL proteins linked to increased risk of arterial disease. Among these, are the phospholipid transfer protein (PLTP) clusterin (CLU) and haptoglobin/haptoglobin related proteins (HP/HPR), thus providing a ground for the reported poor activity of nicotinic acid in CV prevention trials.

The better understanding of HDL function has led to the identification of diseases potentially associated to low HDL-C, such as poor memory or decline in memory in middle-aged adults [34]. More so, the presence or loss of the anti-inflammatory role, potentially affecting the CVD risk reduction [35], led to the understanding of the clear association between HDL-C levels and reduced high sensitivity C-reactive protein (hsCRP) predicting CVD risk reduction [36]. Further, the evidence that increased HDL-C levels over time may lead to a reduction of subsequent CHD, as shown in the 4501 participants in the PHS I (Physician’s Health Study I) prospective study [37], pointed out that antioxidant/anti-inflammatory markers have a clear association with the prognosis of acute MI. The polymorphism of PON-2 (C311S) together with low HDL-C appears to be an additional risk marker [38]. Conversely, of interest is the case of *age-related macular degeneration* (AMD). The disease appears to be associated with elevated apoA-I and HDL-C concentrations leading to a HR of 1.40 (95% CI: 1.20–1.63) and of 1.22 (1.03–1.45), respectively, in Danish individuals in the lowest vs highest quartiles of both apoA-I (130 mag/dL vs 197 mg/dL) and HDL-C (43 mg/dL vs 85 mg/dL) [39].

The correlation between HDL and *insulin secretion/protection from diabetes* has been investigated by numerous authors. Lipid-free and lipid-associated apoA-I and apoA-II increase β-cell insulin secretion and indicate that interventions that raise HDL levels may be beneficial in type 2 diabetes [40]. The apoprotein composition of HDL has drawn particular attention. In the prospective European RISC study in 864 normal volunteers, the HDL subspecies with and without apoC-III showed significant opposite associations with insulin sensitivity at year 3. The highest quintile of HDL containing apoC-III was associated with a 1.2% reduction in insulin sensitivity, whereas the highest quintile of HDL lacking apoC-III was associated with a 1.3% rise [41].

These may be explanatory grounds for the numerous studies indicating not only effective CV prevention by HDL elevation, but also an apparent *raised longevity*, with a mortality risk reduction by 14% for each 10 mg/dL increment in HDL-C before 85 years of age [42]. All these data have, however, still left some doubts on the real association between genetics, HDL-C levels, and risk of acute MI (see above). Testing of different polymorphisms led to inconclusive evidence, such as the observed lack of causality between the polymorphism rs12225230 in the apo A-I locus and CV risk [43]. Although conclusions on the association between genetics of HDL-C and CV risk may apparently go, today, together with the apparent failure of outcome studies with drugs, among others raising HDL-C [44, 45], novel genome-wide association studies may somewhat change opinions. A missense variant in the SID1transmembrane family member 2 (SIDT2) gene (Val636Ile, rs17120425) associates with raised HDL-C levels and reduced CHD risk, apparently by improving HDL function [46].

## Analytical Strategies and Characterization of Subfractions

The difficulty in a definitive conclusion on the arterial benefit of HDL levels and treatment associated rises has prompted several investigations on variables such as the following:

- Analytical methods for defining HDL particles
- Functional properties of HDL as related to cholesterol removal and potential association with HDL-C levels particle distribution

**Fig. 1:**
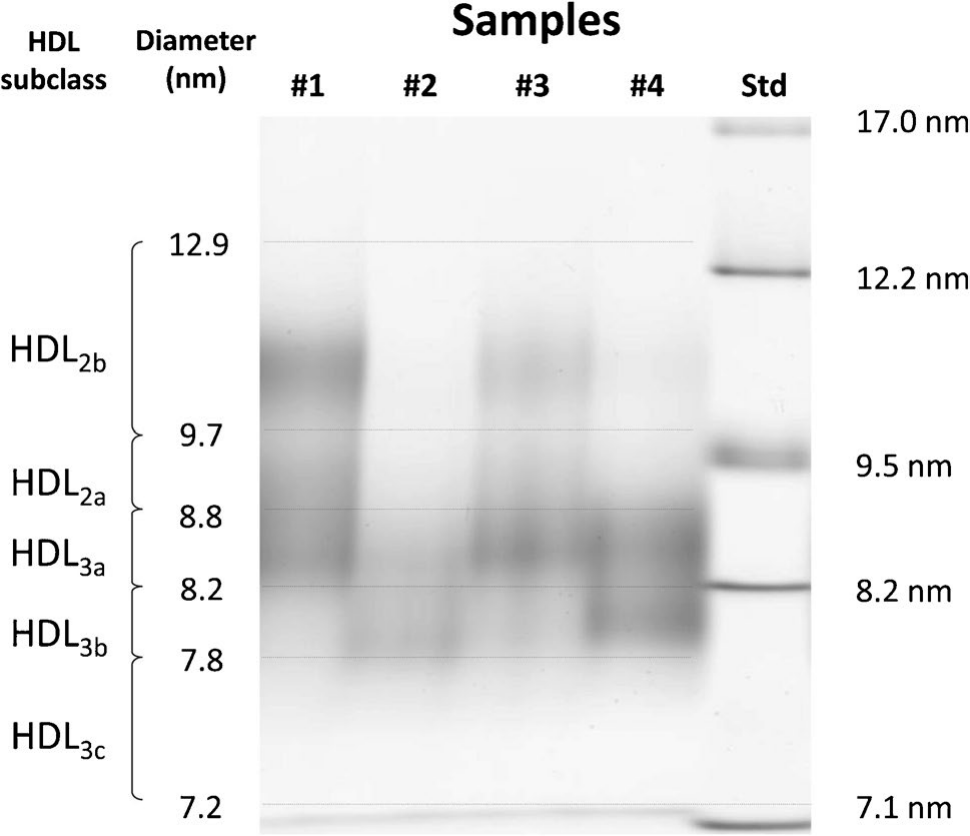
HDL were isolated from plasma by ultracentrifugation at density of 1.21 g/mL and electrophoresed in a 4% to 30% nondenaturing gradient gel. The size distribution was determined by calibration with the use of protein standards (right lane). This procedure resolves 5 distinct sub-classes, although the smallest, HDL3c, is generally present at very low concentrations. ( Reproduced from: Toth PP et al.: J Clin Lipidol 2013, 7(5):484–525, with permission from Elsevier) [47]

Characterization of HDL subfractions has generally relied upon either non-denaturing gradient gel electrophoresis or density gradient fractionation by ultracentrifugation. More recently, subfraction separation has been achieved by two-dimensional gel electrophoresis and nuclear magnetic resonance (NMR) spectroscopy [47]. When separated by particle diameter, in general, five HDL subspecies can be identified by non-denaturing gradient gel electrophoresis (GGE): HDL_3c(7.2–7.8 nm)_, HDL_3b(7.8–8.2 nm)_, HDL_3a(8.2–8.8 nm)_, HDL_2a(8.8–9.7 nm)_, HDL_2b(9.7–12.9 nm)_ (Fig. 1). At this moment, HDL_2b_, the largest HDL, appears to have the strongest inverse correlation with CV risk, as assessed by determination of the carotid intima-media thickness [48]. Density gradient and vertical rotor ultracentrifugation can also well separate the similar particles, allowing to monitor the cholesterol content in each with a between day correlation coefficient ranging between 4 and 9% [49].

The two-dimensional gel electrophoretic technique has gained increasing support because of better visible patterns. Again, five HDL particles are identified: (1) very small (5.6 nm) discoidal precursor HDL containing A-I and phospholipids, with pre-β mobility–*pre-*β *1 HDL*; (2) very small discoidal HDL (7.4 nm) with α mobility containing apo A-I, phospholipids and free cholesterol–*α*_*4*_* HDL*; (3) small spherical HDL (8 nm) with α mobility containing apo A-I, apo A-II, free cholesterol, cholesteryl esters and triglycerides*–* *α *_*3*_* HDL or HDL-M*; (4) medium spherical HDL (9.2 nm) with α mobility, containing apo A-I, apo A-II, free cholesterol, cholesteryl esters and triglycerides, *α *_*2*_* HDL or HDL-L*; (5) very large spherical HDL with α mobility, containing free cholesterol, cholesteryl ester, and triglycerides– *α 1HDL or HDL-VL*.

These separation technologies have allowed to provide a better standardized method to achieve HDL particle separation and correlating these with clinical diagnoses [50]. The epidemiological MESA (Multi-Ethnic Study of Atherosclerosis) study determined instead HDL particle size and protein content by NMR spectroscopy. Levels of the measured HDL-P allowed to detect an excellent correlation between HDL-C and HDL-P and the apparent attenuation by LDL-P levels of the association with HDL-C, not with HDL-P, thus supporting the value of HDL separation by NMR [51]. By NMR spectroscopy, the evaluation of HDL-P provided a clear answer on the conclusion that HDL particle number is a better marker of residual CV risk vs chemically measured HDL-C or apo A-I [52]. The issue of HDL particle number, as assessed by NMR, can provide an indication on the ratio between HDL-cholesterol to HDL protein (HDL-C/P ratio), indicative of cholesterol loading in HDL, and associated with the 5-year progression of carotid atherosclerosis, with a rise in total plaque area [53]. A new case of HDL size enlargement is *pregnancy*. Melchior et al. [54] reported that during late pregnancy 11 different lipoprotein subspecies can be quantified in plasma with NMR by size, including three in the HDL class. The presence of larger HDL particles could be confirmed by tracking phospholipids across lipoproteins by high-resolution gel-filtration chromatography.

The increased risk found in patients with extremely high HDL-C levels in both genders [1, 55, 56] has led to the widely shared conclusion that HDL function, as assessed by cholesterol efflux capacity, is likely the real marker of HDL-associated risk or risk protection. A novel finding related to HDL particle subspecies is the association with incident type 2 diabetes [57]. In the follow-up of the PREVEND study in non-diabetics, those who developed type 2 diabetes showed an inverse association between HDL size and H4P (9.5 nm), as assessed by the NMR platform, and diabetes development. In contrast levels of the small H2P (7.8 nm) showed a positive association. This confirms that larger HDL size and higher H4P associate with a lower diabetes risk, whereas smaller HDL are linked to a higher risk [57]. Anti-diabetic protection by way of HDL may be also exerted via the Hh Smoothened signaling receptor (SMO), protecting HDL during endothelial reticulum stress, and reducing stress-mediated apoptosis. Inhibition of SMO counters the beneficial effects of HDL [58].

An effective approach to HDL characterization and CV risk has been achieved by proteome investigation, based on spectral libraries consisting of 296 protein groups and more than 786 peptidoforms. Performance of this strategy was benchmarked for the detection of proteotype differences between healthy individuals and patients suffering from type 2 diabetes and/or CHD. [59]. Association of these novel technologies allowed to assess unexpected protective mechanisms of HDL, in addition to raised cholesterol efflux, i.e., the inhibition of starvation-induced apoptosis of human endothelial cells and the promotion of maximal respiration of brown adipocytes [60].

## HDL—the Functional Capacity

The most updated views on the protective effect of HDL have led to a better understanding of HDL subparticle distribution and have addressed interest to the functional capacity of HDL to remove arterial cholesterol. The initial observation by Khera et al. [61] showed a strong inverse association between cholesterol efflux capacity (CEC) from macrophages with the prevalence of coronary atherosclerosis, incidence of myocardial infarction, recurrent cardiovascular events, carotid intima media-thickness, and the likelihood of angiographic coronary artery disease, independent of HDL-C and apo A-I levels [62, 63]. Indeed, HDL-C, apo A-I, and total HDL particle concentration only modestly correlate with CEC in most cohorts. CEC is a persistent and robust phenotype that lasts over time, at least for 15 years, as demonstrated in the prospective analysis of the Dallas Heart Study [64]. In terms of specific diseases, the HDL efflux capacity is negatively correlated with type 2 diabetes mellitus and to the incidence of CAD events, independent of other risk factors [65], although evidence of no association was reported in the CODAM Study in subjects with either elevated CVD risk or type 2 diabetes mellitus [66]. Conversely, CEC is impaired in individuals with familial hypercholesterolemia [67], systemic lupus erythematosus [68], non-alcoholic fatty liver disease [69], in women with polycystic ovary syndrome [70], or in individuals with hypogonadism or undergoing hormone affirming therapy [71–73]. In the context of CVD, the evaluation of HDL anti-inflammatory capacity is a functional metric, prospectively providing independent clinical information for the assessment of CVD, as in the case of Framingham Risk Score [74•].

Finally, HDL composition and efflux capacity may be associated with the severity of other diseases, such as COVID-19 infection [75, 76]. Regulation of immunity depends, in fact, on the ability of HDL to influence cholesterol availability in lipid rafts in immune cells, thus modulating toll-like receptors [77, 78]. In the case of the *SARS-CoV-2 virus*, lipid composition is characterized by a rich content of saturated fatty acids such as palmitate, stearate, and arachidonate; palmitoylated proteins appear to have a functional role in viral growth [79]. Most interestingly, linoleate appears to localize within a pocket in the viral spike protein and to interact with remdesivir in reducing viral growth [80].

## Raising HDL with Different Methodologies: Successes and Failures

The apparent causal connection between HDL-C elevations and reduced CV risk prompted several investigations addressed to synthetic drugs and to biotechnological agents, all with a proven HDL-C raising activity. The earliest attempt was by the use of nicotinic acid, previously indicated as effectively reducing CV events in the HATS trial [81]. The two completed trials, AIM-HIGH and HPS2-THRIVE [82, 83], both came up with negative outcomes. While the AIM-HIGH just evaluated coronary patients with low HDL-C, the HPS-2 THRIVE tested, in addition to nicotinic acid, the administration of laropiprant, effectively reducing the disturbing skin vasodilation exerted by nicotinic acid. Both studies provided evidence of raised HDL-C levels, but neither was followed by positive CV outcomes. These trials were however somewhat weakened by problems such as the modest lowering of LDL-C and TG and the mild elevation of HDL-C, somewhat complicating a convincing test of the HDL hypothesis; another hypothesis on the defective activity of nicotinic acid has been reported above (27).

A series of agents with the specific aim of raising HDL are the CETP antagonists, still today the drugs with the best activity on HDL-C raising, in general by over 50%. Depending on their chemical structure, CETP inhibitors that have reached late-stage clinical development are categorized into CETP inhibitors (torcetrapib, anacetrapib, and evacetrapib) and modulators (dalcetrapib). While inhibition of CETP potently raises plasma HDL-C levels, the clinical outcome trials reported generally negative results calling into question the benefit of raising HDL-C [84]. The only exception was with anacetrapib, providing a significant, albeit limited −9% reduction in CV events in a secondary prevention after a median 4.1-year follow-up. Interestingly, the extended follow-up (median 2.2 years) the REVEAL (Randomized Evaluation of the Effects of Anacetrapib through Lipid Modification) study showed an overall 12% RR risk reduction (95%CI 7–17%) corresponding to an absolute 1.8% reduction in major coronary events during the combined median overall follow-up period of 6.3 years [85••]. These findings highlight the need of a sufficient follow-up duration of the RCTs aiming at assessing the CV benefit of lipid-modifying agents.

First-generation CETP inhibitors (torcetrapib, dalcetrapib) were mainly raising HDL-C whereas the next generation CETP inhibitors (anacetrapib, evacetrapib) were also effective in reducing LDL-C and apoB (Table1). An additional mechanism of anacetrapib and other CETP inhibitors of potential benefit, is by way of lipoprotein(a) lowering [86]. A reduction of approximately 35% in lipoprotein(a) could be consequent to a lowered apolipoprotein(a) production after treatment with anacetrapib [87]. This observation was confirmed in the larger phase III clinical trial including 30,449 adults with CV disease, in whom the addition of 100 mg/day of anacetrapib to intensive statin over a median follow-up of 4.1 years reduced mean lipoprotein(a) by 25% [88]. Another CETP inhibitor, evacetrapib, 500 mg as monotherapy or in combination with statin, reduced lipoprotein(a) by 30–40% over a period of 12 weeks in 393 patients [89], and a similar activity was displayed by obicetrapib (TA-8995) (see below).

**Table 1:** Lipid percentage changes upon treatment with CETP inhibitors

Drug	Torcetrapib	Dalcetrapib	Evacetrapib	Anacetrapib	Obicetrapib
Clinical trials	ILLUMINATE [141]	Dal-OUTCOMES [142]	ACCELERATE [143]	REVEAL [88]	ROSE [96]
Dose	60 mg	600 mg	130 mg	100 mg	5 mg and 10 mg
No. of patients	15,067	15,871	12,092	30,449	120
LDL-C (mg/dL)	− 24.9%	Minimal effect	− 31.1%	− 41%	− 36% (Friedwald) with 5 mg − 43.4% (Friedwald) with 10 mg
apoB (mg/dL)	NA	Minimal effect	− 15.5%	− 18%	− 22.6% with 5 mg − 27.2 with 10 mg
HDL-C (mg/dL)	+ 72.1%	Range: + 31–40%	+ 133.2%	+ 104%	+ 124% with 5 mg + 156% with 10 mg
Non-HLD-C (mg/dL)	NA	NA	NA	− 18%	− 34.3% with 5 mg − 39.3% with 10 mg

ACCELERATE, assessment of clinical effects of cholesteryl ester transfer protein inhibition with evacetrapib in patients at a high-risk for vascular outcomes; Dal-OUTCOMES, a study of the effect of dalcetrapib on atherosclerotic disease in patients with coronary artery disease; ILLUMINATE, a study examining torcetrapib/atorvastatin and atorvastatin effects on clinical CV events in patients with heart disease; REVEAL, randomized evaluation of the effects of anacetrapib through lipid-modification; ROSE, randomized study of obicetrapib as an adjunct to statin therapy. This is a phase 2 study enrolling 120 patients allocated to 3 arms: placebo (*n* = 40), obicetrapib 5 mg (*n* = 40) and 10 mg (*n* = 40). Adapted from Ferri N, et al. Pharmacol Res 2018, 128:29–41, with permission from Elsevier [44]

*Apo* apolipoprotein, *CETP* cholesteryl ester transfer protein, *HDL* high-density lipoprotein, *LDL* low-density lipoprotein, *NA* not applicable

In the era of genome-wide association study, data from the Dal-GenE trial (NCT02525939), testing the CV preventive effect of dalcetrapib in acute coronary syndrome patients—carriers of the AA genotype at rs1967309 in the *ADCY9* gene—are eagerly awaited [90] also in consideration of the failure of the same genotype in demonstrating any beneficial effect upon evacetrapib [91] or anacetrapib [92] treatments. A comparative MR study evaluating in parallel CETP protein concentrations with those of proprotein convertase subtilisin/kexin type 9 (PCSK9) [93] found significant between-compound heterogeneity in effects on lipids, blood pressure, and clinical outcomes. On-target CETP inhibition, assessed through MR, appeared to reduce the risk of CHD, heart failure, diabetes, and chronic kidney disease, whereas the risk of AMD was raised, possibly providing an explanation to the observed raised risk in subjects with elevated A-I levels and HDL-C [39]. In contrast, lower PCSK9 concentrations appeared to reduce the risk of CHD, heart failure, atrial fibrillation, chronic kidney disease, multiple sclerosis, and stroke, potentially raising the risk of Alzheimer’s disease and asthma. Similar findings were recently reported in subjects with CETP deficiency in the general population [94], showing lower risk of CV mortality, ischemic heart disease, MI, peripheral artery disease and vascular dementia with, however, a markedly higher risk of AMD (HR, 2.33; 95% CI, 1.63–3.30).

The overall lack of success of CETP antagonists, apart from anacetrapib, most likely because of its better activity on non-HDL-C levels, led to a loss of interest in these agents. However, at least one product, obicetrapib (TA-8995), had shown positive results from a phase 2 study in patients with LDL-C elevation and reduced HDL-C (0.8–1.8 mmol/M) [95]. Obicetrapib reduced LDL-C by 27.4% with a 1 mg daily dose, and up to 45.3% in those given the 10 mg dose, with an additive effect to statins. HDL-C levels went up by 75.8% with the 2 mg dose and by + 179% with 10 mg. Lipoprotein(a) was significantly reduced by 33.4% (24.4–42.5%) [95]. The product was left aside for several years but recently the New Amsterdam company acquired it from Amgen and completed a randomized clinical study on 5 or 10 mg/day with or without 10 mg ezetimibe in patients with mild dyslipidemia. Data from this Phase II trial, ROSE (Randomised study of Obicetrapib as an Adjunct to Statin Therapy), categorized the 120 participants (median LDL-C of 88 mg/dL at baseline) to receive either a 5 mg or 10 mg dose of obicetrapib or placebo for eight weeks. The drug was analyzed as a single agent and along with ezetimibe, as an adjunct to high-intensity statin therapy. Compared with placebo, obicetrapib led to a dose-dependent lowering of LDL-C (up to 50.8%), a lowering of apoB (up to 29.8%) and non-HDL-C (up to 44.4%) and raising of HDL-C (up to 165%). There were no major side effects associated with the new agent [96]. In January 2022, the 52-week BROADWAY phase 3 trial (NCT05142722), testing the efficacy of obicetrapib in adults with heterozygous familial hypercholesterolemia (HeFH) and/or established atherosclerotic cardiovascular disease, has enrolled the first patient. The company has planned to randomize 2400 patients to treatment with either placebo or 10 mg obicetrapib q.d.. The primary objective of the trial is to evaluate the effects of treatment on LDL-C levels, with the results of the study expected in Q1 2024.

It thus appears that CETP antagonism, even with drugs with different mechanisms and even testing for associated genetic abnormalities, as above noted, up to now have not provided a convincing CV benefit. The efficacy of HDL on different vascular and non-vascular endpoints has however recently indicated that CETP antagonism, by maintaining elevated HDL-C levels, may reduce *severity of sepsis* in high-risk conditions [97]. CETP gain-of-function variants in a 7-cohort meta-analysis were in fact associated with an increased risk of acute sepsis mortality, whereas a genetic score for reduced function was associated with a 25–40% reduced mortality. In mice transgenic for CETP, anacetrapib showed a significantly increased sepsis survival.

The case of fibrates is still open. Although it has been generally noted that raising HDL-C with fenofibrate will not lead to CV benefit [98], this may not be the case. While the gemfibrozil secondary prevention trial VA-HIT (the Veterans Affairs Cooperative Studies Program High-Density Lipoprotein Cholesterol Intervention Trial) [99] had shown that a 7.5% increase of HDL-C leads to a 22% reduction of non-fatal MI and CHD deaths and a 10% reduction of all-cause mortality, the HDL-C rise after bezafibrate in the BIP (Bezafibrate Infarction Prevention Trial) study [100] failed to show benefit in patients with normal triglycerides but led to a 40% reduction in hypertriglyceridemics with HDL-C < 35 mg/dL [101]. The FIELD study with fenofibrate, in diabetics with a total cholesterol to HDL-C ratio > 4.0 or TG > 88.6 mg/dL, also failed to show a reduction in composite of death and non-fatal MI in the 5-year follow-up, but the secondary composite endpoint of total CV events at 5 years (as generally reported in the statin trials) indicated a significant reduction (− 10%, *p* < 0.035 with fenofibrate). Similar results were reported in the ACCORD (Action to Control Cardiovascular Risk in Diabetes) trial [102] where again in the subgroup with TG ≥ 204 mg/dL and HDL-C ≤ 34 mg/dL, the reduction in the primary endpoint was 31%. In general, by pooling all endpoints in the low HDL-/high TG patients, CV benefit data were not remarkably different from those found in the statin trials (Table 2). In the case of fenofibrate, the reduction of CV events was significantly associated with a PPARα variant (rs6008845, C/T) on chromosome 22 [103].

**Table 2:** Comparison of the ACCORD Lipid Trial to earlier fibrate trials

Trial	Drug	Primary endpoint: entire cohort	Lipid subgroup criterion	Primary endpoint: subgroup
HHS [144, 145]	Gemfibrozil	− 34%; *p* = 0.02	TG > 200 mg/dL LDL-C/HDL-C > 5.0	− 71%; *p* = 0.005
BIP [100]	Bezafibrate	− 7.3%; *p* = 0.24	TG ≥ 200 mg/dL	− 39.5%; *p* = 0.02
FIELD [146, 147]	Fenofibrate	− 11%; *p* = 0.16	TG ≥ 204 mg/dL HDL-C < 42 mg/dL	− 27%; *p* = 0.005
ACCORD [102]	Fenofibrate	− 8%; *p* = 0.32	TG ≥ 204 mg/dL HDL-C ≤ 34 mg/dL	− 31%

Percent reduction in primary cardiovascular endpoint in the entire cohort versus subgroups entering the trials with high low HDL-C and/or high triglyceride (Lipid Subgroup). ACCORD, Action to Control Cardiovascular Risk in Diabetes; BIP, Bezafibrate Infarction Prevention; FIELD, Fenofibrate Intervention and Event Lowering in Diabetes; HHS, Helsinki Heart Study. From: ACCORD Study Group, Ginsberg HN, et al. 2010, 362(17):1563–1574. Copyright © 2010 Massachusetts Medical Society. Reprinted with permission from Massachusetts Medical Society) [102]

Since in individuals with hypertriglyceridemia and low HDL-C treated with fibrates, the CV risk reduction appears not to differ from that found with statins in patients with elevated cholesterol, it is of interest to wait for the data on pemafibrate, a novel fibrate derivative with very low daily dosages, being tested in the PROMINENT (Pemafibrate to Reduce Cardiovascular Outcomes by Reducing Triglycerides in Patients with Diabetes) on selected coronary patients with hypertriglyceridemia [104, 105].

HDL function may be also modulated by *dietary approaches*. Hernáez et al. [106] investigated the effect of a Mediterranean diet from the PREDIMED (Prevención con Dieta Mediterránea) study. Two dietary varieties were investigated, one enriched with virgin olive oil (VOO) and the other with nuts: both raised the CEC relative to baseline in addition to a raised vasodilating capacity. The VOO intervention decreased CETP activity and increased HDL ability to esterify cholesterol, paraoxonase-1 arylesterase activity, and HDL vasodilatory capacity [106].

An improved reverse cholesterol transport (RCT) was also found in mice following angiopoietin like protein-3 (ANGPTL3) inhibitory treatment, apparently improving RCT, while reducing HDL-C levels [107]. Loss-of-function (LOF) mutations of ANGPTL3 in humans result in a condition called familial combined hypobetalipoproteinemia (FHBL2) characterized by low levels of all lipoprotein classes (VLDL, LDL and HDL) [108] with no atherosclerosis risk. Reduced levels of HDL-C and of LDL-C could be attributed to the activation of endothelial lipase [109]. However, regardless of the effect on HDL-C, treatment with *Angptl3* ASO significantly enhanced RCT, thus pointing out to a CV protective activity of ANGPTL3 antagonism, now available in injectable form for human use [110].

## Direct Use of HDL

The inconstant activity of HDL in a variety of in vitro systems, and the possibility that, even in the face of raised levels, no CV benefit may occur, has stimulated interest in the direct use of HDL, be it as synthetic lipoproteins with biotechnologically engineered apo A-I or genetic variants such as the apo A-I_Milano_. This approach provided early very stimulating reports from animal studies [111]. Human evaluation of a single dose or reconstituted human HDL (rHDL) vs saline infused into patients with peripheral artery disease undergoing femoral atherectomy, reduced plaque lipids and vascular adhesion molecules and macrophage cell size compared with the placebo group [112]. This was preceded by a major clinical intravascular ultrasound (IVUS) study of recombinant apo A-I_Milano_ (five injections of 15–45 mg/kg). The 4.2% decrease from baseline of total coronary plaque volumes in this study [113] induced a considerable excitement in the medical and industrial community. The development company (Esperion Therapeutics) was acquired by Pfizer, allowing a more extensive investigation of the protein’s activity. Unfortunately, a newer preparation of A-I_Milano_ tested in coronary patients led to severe allergic reactions with one casualty.

**Fig. 2:**
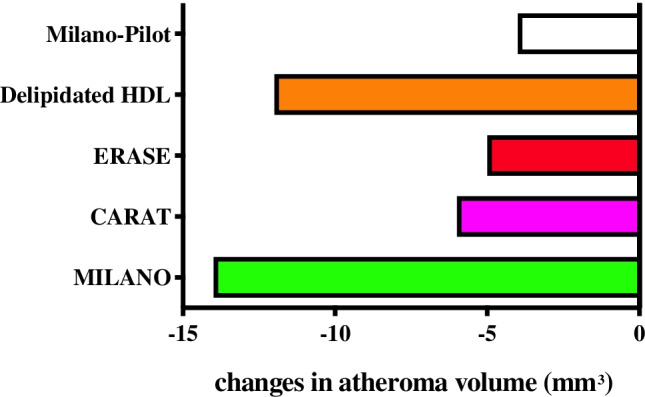
Intravascular ultrasound studies of HDL (high-density lipoprotein) mimetics. Change in atheroma volume infusing HDL mimetics containing apoA-I_Milano_ in 2003 (MILANO), wild-type apoA-I and sphingomyelin (CARAT), wild-type apoA-I (ERASE), autologous delipidated HDL (Delipidated HDL), and apo A-I_Milano_ in 2016 (MILANO-PILOT). CARAT, The CER-001 Atherosclerosis Regression Acute Coronary Syndrome Trial [140]; ERASE, Effect of rHDL on Atherosclerosis—Safety and Efficacy [114]. ( Reproduced from: Sirtori CR, et al. Ann Med 2019, 51(7–8):345–359, reprinted by permission of Taylor & Francis Ltd, https://www.tandfonline.com/) [2]

In more recent years, other products have been tested in human trials mainly with the IVUS method, i.e., in addition to A-I_Milano_ dimer, preparations of normal human A-I complexed in different formulations, e.g., testing addition of phosphatidylserine (PS) and sphingomyelin (SM), in contrast to the 1-palmtoyl-2-oleyl-phsphocholine (POPC) used in the A-I_Milano_ trial. SM and PS appear to provide some benefit in terms of anti-inflammatory properties [114]. Overall intravascular ultrasound studies of HDL mimetics containing apo A-I_Milano_ or wild-type A-I [115] indicated an arterial benefit but with some unclear issues as to the final conclusions (Fig. 2). The most recent investigations proved, in fact, somewhat questionable. The same research group responsible for the earlier study on A-I_Milano_, now MDCO-216, tested 120 patients randomized to either placebo (*n* = 60) or MDCO 216 (20 mg/kg, *n* = 52) for 5 weekly infusions but, differently from the original trial, they did not report a significant benefit in terms of reduced progression in the IVUS images [116]. In this study, there were no changes in lipoprotein profile, differing from the previous study and, in addition, hsCRP was raised, thus possibly indicating some structural features that may be different from the original product used in the early clinical report [117]. Other studies with A-I mimetics have provided unclear findings [118].

The case of CER-001, a negatively charged lipoprotein particle with human recombinant apo A-I, and two natural phospholipids: sphingomyelin (Sph) and dipalmitoylphosphatidylglycerol (DPPG), provided interesting insights into the potential activities of A-I mimetics. The product induced some regression of coronary atherosclerosis in the patients with more extensive plaque burdens [119], but it failed to show coronary benefit in the large IVUS-based CARAT (CER-001 Atherosclerosis Regression Acute Coronary Syndrome Trial) study [120]. Interestingly, CER-001 appears to have a therapeutic target in inherited lecithin cholesterol acyltransferase (LCAT) deficiency as shown in an animal model [121] and in a series of patients [122], apparently by way of lipoprotein remodeling and particularly dramatic reduction of LpX. This beneficial effect occurs earlier than predictable and may offer a novel way of treatment for this serious condition [123].

Coronary studies in the most recent series, all investigated a few infusions and patients with optimal drug treatment, i.e., different from early study by Nissen on A-I_Milano_ [116]. Several questions need to be answered: are five infusions enough or should the agent be tested in conditions of maximal drug treatments aimed to arterial benefit? Are doses adequate? Some authors believe that based on comparative activities the doses used in the most recent trials were way too low [124]. There is finally need for some newer methods for assessing coronary wall thickness by non-invasive technologies. Preliminary observations were reported by using an ultrasound method measuring wall thickness of the left main coronary [125].

An exciting development has been brought about using an oral formulation of A-I_Milano_ by using genetically modified rice plants. This transgenic “rice milk” is apparently not degraded in the intestine and remains in the dimeric form, thus being active in markedly reducing the extent of atherosclerotic plaques compared to E-KO mice receiving normal rice milk [126]. Higher doses appear to improve diet induced liver steatosis. Aside from this indication, involving classical approaches to arterial plaques, apo A-I and A-I mimetics [2], in particular A-I_Milano_, have been shown to be effective on animal models of stent biocompatibility [127] and of experimental heart failure (HF), by using models of reduced ventricular ejection fraction [128]. These findings have prompted planning some early clinical evaluations of HDL therapy in HF patients.

Finally, among mimetic short chain peptides, the D-4F has received the largest interest having some of the anti-inflammatory properties of HDL, improving vasodilatation and inhibiting atherosclerosis in mice models [129]. Unfortunately, the product turned out to be poorly absorbed and with minimal effectiveness in man [130].

One final application of HDL could be in *tumors*. HDL reduce cancer cell content of cholesterol, overall rewiring cholesterol homeostasis, reducing oxidative stress and the levels of pro-inflammatory molecules in cancer cells and in the tumor microenvironment [131]. Reducing lipoprotein uptake and stimulating cell cholesterol efflux could represent a novel adjuvant strategy in hormone dependent cancer management [132]. In prostatic cells, HDL blunted oxidative stress and reduced proliferation, with a role for both the protein and phospholipid components [133]. HDL binds the scavenger receptor class B type I [134] highly expressed in tumor cells thus making HDL suitable for delivery of therapeutic agents in cancer treatment [135].

## Conclusions

HDL in 2022 is still an open field with a steadily growing number of scientific contributions. The role of HDL-C concentrations in 2022 remains that of a biomarker in CVD risk prediction [136] as confirmed in a large number of clinical investigations and as supported by mechanistic studies on cholesterol efflux and other properties, such as anti-inflammatory and possibly antidiabetic [137]. HDL-C elevations may not be, however, appropriate targets of treatment, also in view of the apparent U-shaped relationship between HDL-C elevations and CV risk and the still unsettled support from clinical studies with HDL raising drugs and HDL mimetics. While reanalysis of the studies on fibrates probably indicates the benefit of these in raising HDL-C and consequently reduce CV events in appropriate patients, the opportunity of having HDL mimetics should not be left aside. Potentially the oral formulation of A-I_Milano_ may encourage further evaluations. Interventions in diabetes, HF and cancer are other areas that have not been extensively explored. Future therapeutic strategies should focus on optimizing HDL function in the right patients at the optimal time in their disease course. A framework is needed to help search in clinical communities, as well as funding agencies and stakeholders, to obtain insight into current thinking on these topics [138]. Particularly young scientists should recall that the statin era started over 30 years ago and this new field of investigation is far younger and perhaps more promising. It is likely not the time “to call the plumber” [139].
